# Comprehensive cohort study: computer tomography-guided high-dose rate brachytherapy as metastasis-directed therapy for liver metastases from colorectal cancer in repeat oligoprogression

**DOI:** 10.1007/s11547-025-01988-y

**Published:** 2025-03-13

**Authors:** Mateusz Bilski, Katarzyna Korab, Magdalena Orzechowska, Julia Ponikowska, Paweł Cisek, Barbara Alicja Jereczek-Fossa, Jacek Fijuth, Łukasz Kuncman

**Affiliations:** 1 Department of Brachytherapy, Saint John’s Cancer Center, Lublin, Poland; 2 Department of Radiotherapy, Medical University of Lublin, Lublin, Poland; 3 Department of Radiotherapy, Saint John’s Cancer Center, Lublin, Poland; 4 Department of Medical Physics, Saint John’s Cancer Center, Lublin, Poland; 5 Department of Molecular Carcinogenesis, Medical University of Lodz, Lodz, Poland; 6 Division of Radiation Oncology, European Institute of Oncology IRCCS, 20141 Milan, Italy; 7 Department of Oncology and Hemato-Oncology, University of Milan, Milan, Italy; 8 Department of Radiotherapy, Medical University of Lodz, Lodz, Poland; 9 Department of External Beam Radiotherapy, Copernicus Memorial Hospital in Lodz Comprehensive Cancer Center and Traumatology, Pabianicka 62, 93-513 Lodz, Poland

**Keywords:** Interventional radiotherapy (I-RT), Brachytherapy, Oligometastatic disease, Liver cancer, Liver metastases, Colorectal cancer, Liver radiotherapy, Metastasis directed therapy, Computer tomography (CT)-guided high-dose rate (HDR) brachytherapy (BRT)

## Abstract

**Purpose:**

The standard treatment for oligometastatic colorectal cancer includes systemic therapy, with surgery and metastasis-directed therapy as options. The optimal strategy, especially for repeat oligoprogression (rOP), remains unclear. We report outcomes of liver computer tomography-guided high-dose rate brachytherapy (CT-BRT) in this setting.

**Methods:**

This retrospective cohort study included colorectal cancer patients with liver-only oligoprogression during systemic therapy, meeting criteria of up to 5 liver metastases, CT-BRT eligibility, and ECOG status ≤ 2. Patients were followed for local response, progression-free survival (PFS), overall survival (OS), and toxicity. Response, according to RECIST 1.1, was initiated 6 months post-CT-BRT.

**Results:**

A total of 262 metastases were treated in 127 patients, with 67.7% receiving third-line or later systemic therapies. One to four liver metastases were found in 29.1%, 42.5%, 21.2%, and 7.1% of patients, respectively, with a median volume of 128 cm^3^. A median of 3 applicators was used, with CT-BRT doses of 15 Gy, 20 Gy, and 25 Gy given to 29.9%, 41.7%, and 28.3% of patients. At 6 months complete response occurred in 3.1%, progressive disease in 23.6%, partial response in 19.7%, and stable disease in 53.5%. Median PFS was 9 months, median OS was 16 months, with 1-year and 2-year OS rates of 65% and 16%, respectively. Liver-only metastases and objective response were associated with longer PFS. The G3 toxicity was 4.0%; no events > G3 were reported.

**Conclusions:**

This largest study documents favorable outcomes of liver CT-BRT for rOP, establishing this method as a viable option in this indication.

**Supplementary Information:**

The online version contains supplementary material available at 10.1007/s11547-025-01988-y.

## Introduction

Metastasis-directed therapy (MDT) in oligometastatic disease (OMD) has been proven to be a valid addition to the standard of care for selected patients, leading to prolongation of progression-free survival (PFS) and/or overall survival (OS) in many cancers [1–3]. Liver metastases develop in 35–80% of colorectal cancer (CRC) patients [4–6]. Surgical management of liver metastases can be proposed in only 10–20% of patients [4, 7]. The remaining 80–90% of patients with liver OMD are considered on multidisciplinary tumor boards for other ablative techniques [5].

Radiation therapy, particularly stereotactic body radiotherapy (SBRT), is an effective treatment option for liver metastases, demonstrating excellent 1-year local control (LC) rates ranging from 87 to 97.3%, with a favorable safety profile, as grade ≥ 3 toxicity occurs in only 2.6–3.7% of patients [8, 9]. However, there are limitations involving breathing movements of the liver, with very more generous planning target volumes (PTV) margins are often needed for external beam radiotherapy (EBRT) modalities. Even with modern machines like MR-LINACs and 4D-MRI guided SBRT, substantial mid-position drifts can occur, which stresses the need for intra-fraction motion management strategies to increase the precision of treatment delivery [10].

Taking into consideration the prolongation of the patient’s PFS and OS, with modern systemic therapy, more frequent use of MDT as a form of reirradiation is often considered [11]. In that setting, the dosimetric evaluation of doses in uninvolved livers and other organs at risk (OARs) must be closely considered. Interventional computer tomography-guided high-dose rate brachytherapy (CT-BRT) has been proven to be effective, with 1y LC of 70–100% [12, 13]. This minimally invasive radiotherapy method also has an excellent safety profile with minor and major complication rates of 6 and 4%, respectively [12, 14, 15].

In 2020, experts from European Society for Radiotherapy and Oncology (ESTRO), and the European Organisation for Research and Treatment of Cancer (EORTC) published a recommendation on a division of OMD. Nine clinical scenarios were proposed for implementation in clinical trials [16]. We have selected patients with repeat oligoprogression (rOP) of CRC only, for which we have used MDT for liver metastases performed with CT-BRT. Our study aimed to evaluate oncologic outcomes and toxicity profiles of liver CT-BRT and to facilitate future comparison of individual studies in that specific OMD clinical scenario in CRC, as well as to enable validation of the OMD classification proposed by ESTRO/EORTC in the future [17].

## Materials and methods

**Fig. 1:**
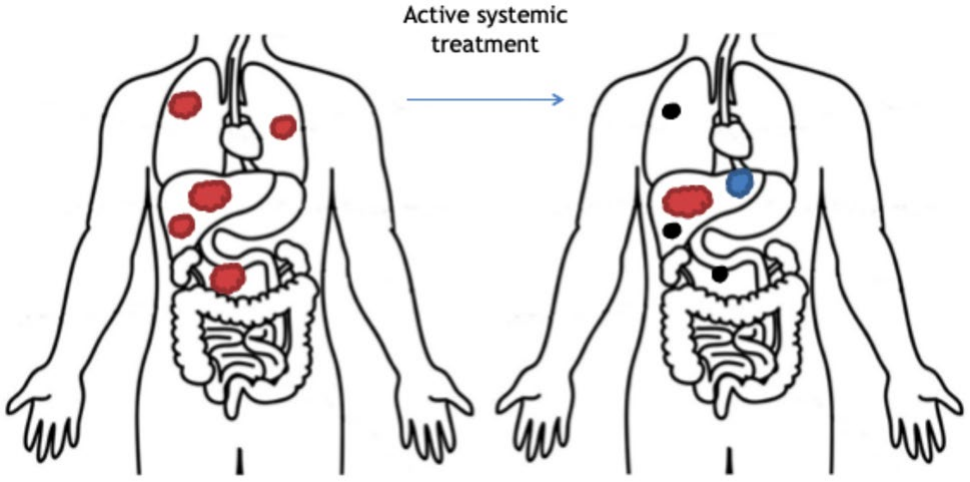
A precise explanation of the clinical scenario of repeat oligoprogression for patients is included in the analysis. Previous diagnosis of Oligometastatic disease (OMD) with up to 5 metastases in different localizations (left image), Diagnosis of growing or regrowing (red) or new metastases (blue) only in patients’ liver during active systemic therapy (right image)

The medical records of patients with CRC and liver metastases treated CT-BRT at the first author’s institution from 2015 to 2022 were retrospectively reviewed. For this analysis, only the patients with rOP, diagnosed CT and/or positron emission tomography/CT (PET/CT), in the liver were included. The rOP was defined according to ESTRO/EORTC recommendations following the rules: (1) previously present oligometastatic disease followed by local treatment or systemic treatment or both, (2) being under active systemic therapy, (3) oligoprogression defined as a diagnosis of new and growing or regrowing metastases in the maximum number of 5 as shown in Fig. 1. Other inclusion and exclusion criteria are presented in Table 1.

**Table 1:** General inclusion and exclusion criteria used for the qualification to computer tomography (CT)-guided high-dose rate brachytherapy (BRT) procedure

Additional inclusion criteria:	Exclusion criteria
WHO score < 3	Tumor location that makes it impossible to place the applicator
Tumor diameter < 10 cm	
Number of metastases ≤ 5	
Creatinine level < 2 mg/dL	Inflammation in the abdominal cavity
HGB > 8 mg/dL	
WBC > 2000/mm^3^	
NEU > 1500/mm^3^	Close proximity to critical structures that prevent the planned dose from being achieved
PLT > 50,000/mm^3^	
INR < 1.5	
ALT, AST, BIL total < 2.5 × upper limit of normal	

At the time of diagnosis of rOP (and CT-BRT) no patient had switched the line of systemic treatment. The change of systemic therapy line was allowed after subsequent progression.

The study was conducted following the latest version of the Declaration of Helsinki. The local Ethics Committee approved the study (approval no. LIL-KB-20/2014). Because of the retrospective character of the study, no patient agreement was mandatory. We have followed the Strengthening the Reporting of Observational Studies in Epidemiology (STROBE) guidelines, and the checklist is presented in Supplementary Materials 1.

### CT-BRT procedure explanation

**Fig. 2:**
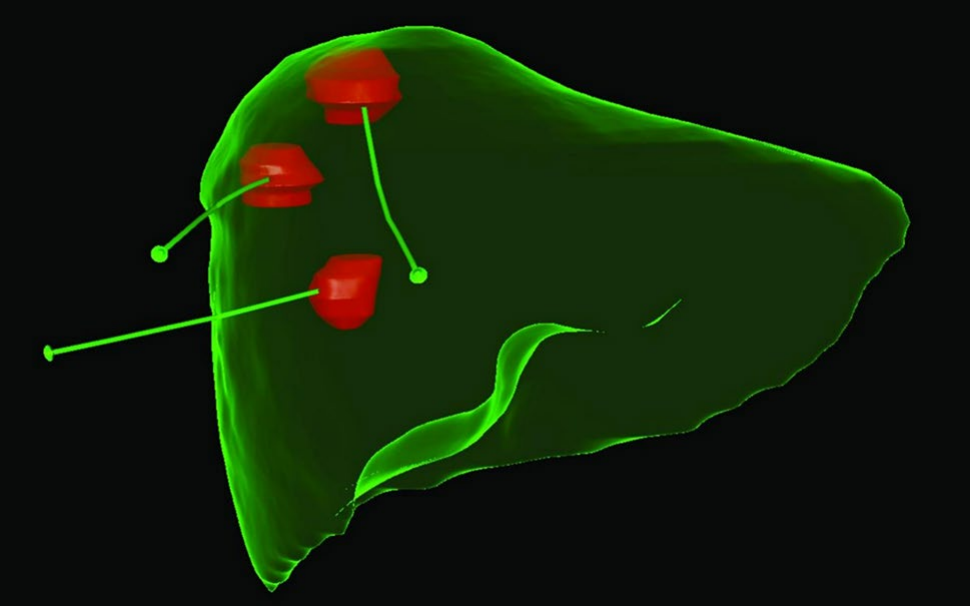
Exemplary 3D view of one of selected patients with three different metastases treated during one procedure

**Fig. 3:**
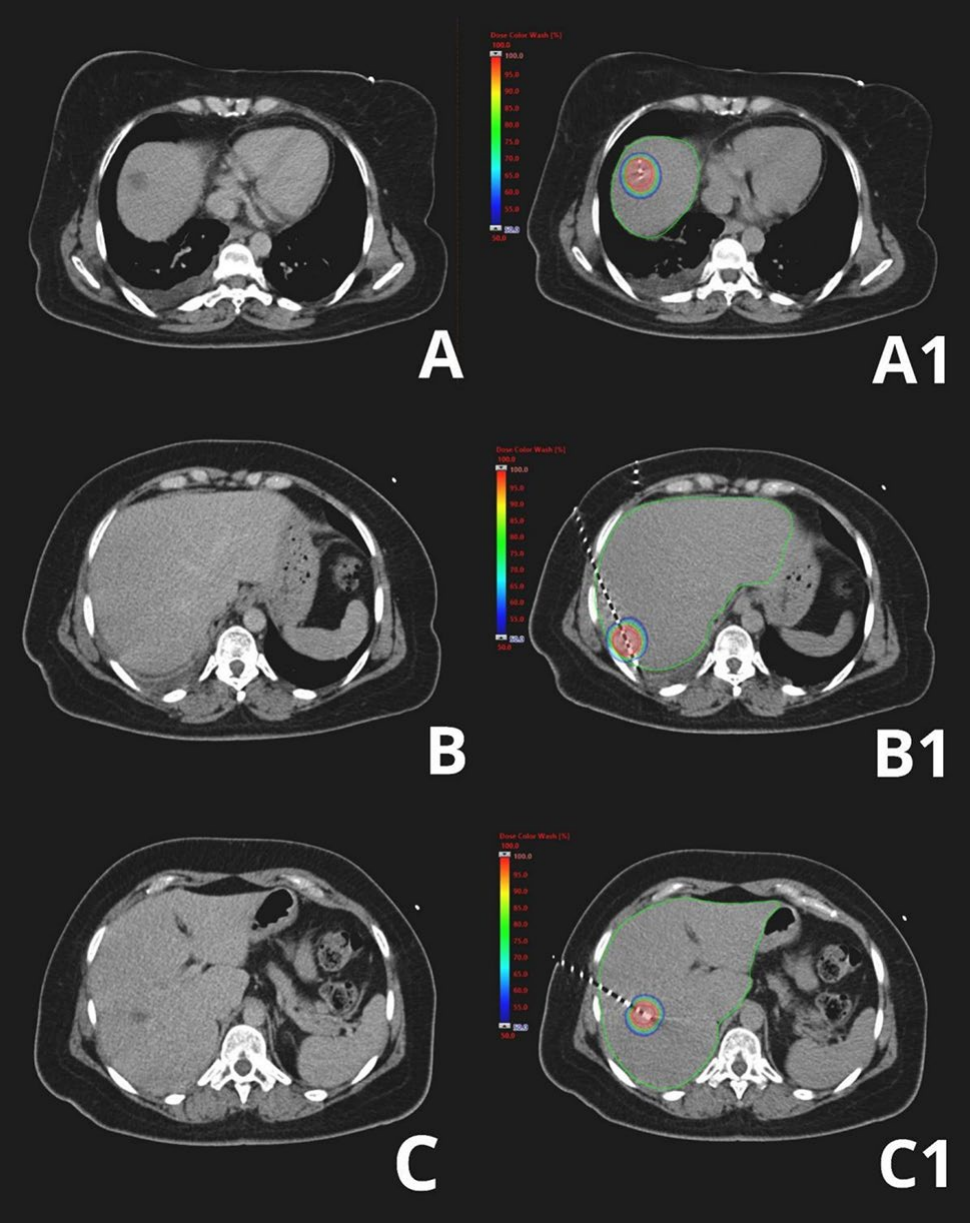
Example of one of the selected patients treated with computer tomography (CT)-guided high-dose rate brachytherapy (BRT): diagnostic CT images of three different metastases before procedure (**A**, **B**, **C**), and dose distribution profiles (**A1**, **B1**, **C1**)

All selected patients received general anesthesia. After contrast administration, the simulation began with the radiation oncologist (RO) using a CT to match the upper and lower borders of the tumor. These borders were drawn on the patient’s skin before the application started. The RO then performed direct applications using 200 or 320-mm long applicators (Varian, Inc.), and a 32-slice CT scanner with real-time fluoroscopic imaging was used (Fig. 2). After the manual part of the procedure, a CT scan with a 1.5–3 mm slice thickness was done. The RO delineated the gross tumor volume (GTV) using fusion with diagnostic CT and/or MRI and/or PET/CT. No additional margins were applied to form a clinical target volume (CTV) or PTV. The OARs were contoured according to metastasis localization, and specified dose constraints were applied (Supplementary Materials 2 Table S1). The source step was set to 5 mm. In most cases, dose volume optimization was carried out using inverse planning as a starting point for manual optimization. All patients were planned using the BrachyVision planning system version 10 or 16 (Varian Medical Systems, Inc.), example treatment plan is shown on Fig. 3. Dose calculation and prescription were performed using the TG-43 algorithm, with a single fraction of 15 Gy, 20 Gy, or 25 Gy administered to all patients. The target dose was 25 Gy; however, lower doses were administered due to dose constraints in the organs at risk (OARs), primarily the gastrointestinal tract (small intestine, duodenum, and stomach). Conversely, no dose restrictions were implemented due to the extensive volume of lesions. Treatment plans were delivered using BRAVOS or GammaMed iX HDR iridium 192 after loaders (Palo Alto, USA). Previous publications described the rules of CT-BRT applications, which typically involved a 3-day hospital stay, with CT-BRT performed on the second day under anesthesia, and the entire procedure lasting 30 min [18–20].

The first response evaluation, with chest, abdomen, and pelvis CT, was performed after 3–4 months from the CT-BRT procedure and was done according to RECIST 1.1 criteria. The second evaluation was performed after 6–7 months and was selected for response evaluation inside the liver. Before that time, there was a possibility of misinterpretation of diagnostic tests, mainly in favor of progression (PD) diagnosis, because of frequent central necrosis, hypointense rim, inflammation, and/or edema present inside and around metastasis [21, 22]. If the PD was present during the first and second diagnostic evaluation, it was counted from the first diagnostic scan. Further evaluation was done regularly at 3–4 months intervals with the same diagnostic tests.

The primary endpoint was 1- and 2-year PFS and 1- and 2-year OS. The OS was determined from the date of the CT-BRT treatment to the time of death or last follow-up (FU). The PFS was calculated from the date of CT-BRT to local or distant progression (whichever occurs first) or last FU. Secondary endpoints were the evaluation of objective response rate (ORR), disease control rates (DCR), and potential toxicity using Common Terminology Criteria for Adverse Events 5.0. Acute toxicity was defined as those appearing within three months of CT-BRT, while late toxicity was defined as those appearing after that period.

### Statistics

Baseline variables describing the patients characteristics were summarized with median and range for continuous variables and counts accompanied by percentages for categorical variables. Survival analysis was performed with the Kaplan–Meier curves and log-rank test for comparison, followed by the uni- and multivariate Cox proportional-hazards regression model to identify OS and PFS prognosis-relevant variables. All patients lost from follow-up were censored during analyses. Finally, the optimal cutpoint determination algorithm (EvaluateCutpoints, cutp algorithm) was employed to differentiate patients’ prognosis according to continuous variables such as the number of metastases or total volume of all lesions [23]. All analyses were performed using R v. 4.2.0. (Lucent Technologies, USA).

## Results

**Fig. 4:**
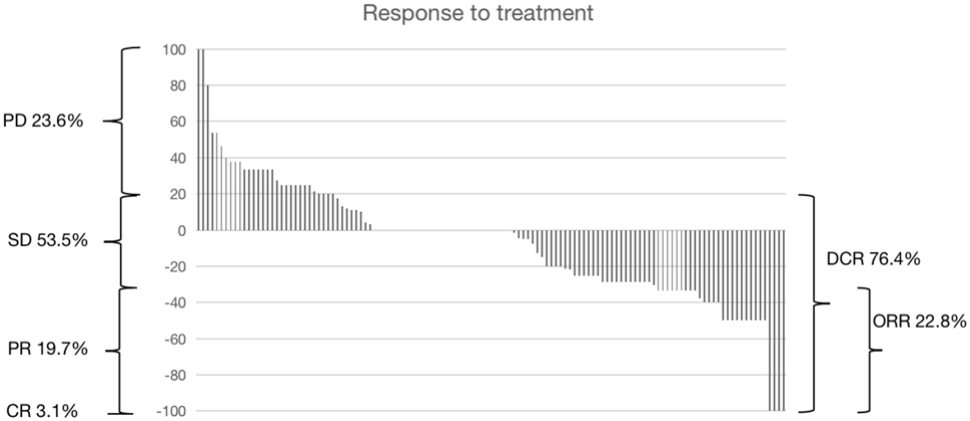
Proportion of Complete Response (CR), Partial Response (PR), Stable Disease (SD), and Progressive Disease (PD) According to Response Evaluation Criteria in Solid Tumors (RECIST) 1.1

**Fig. 5:**
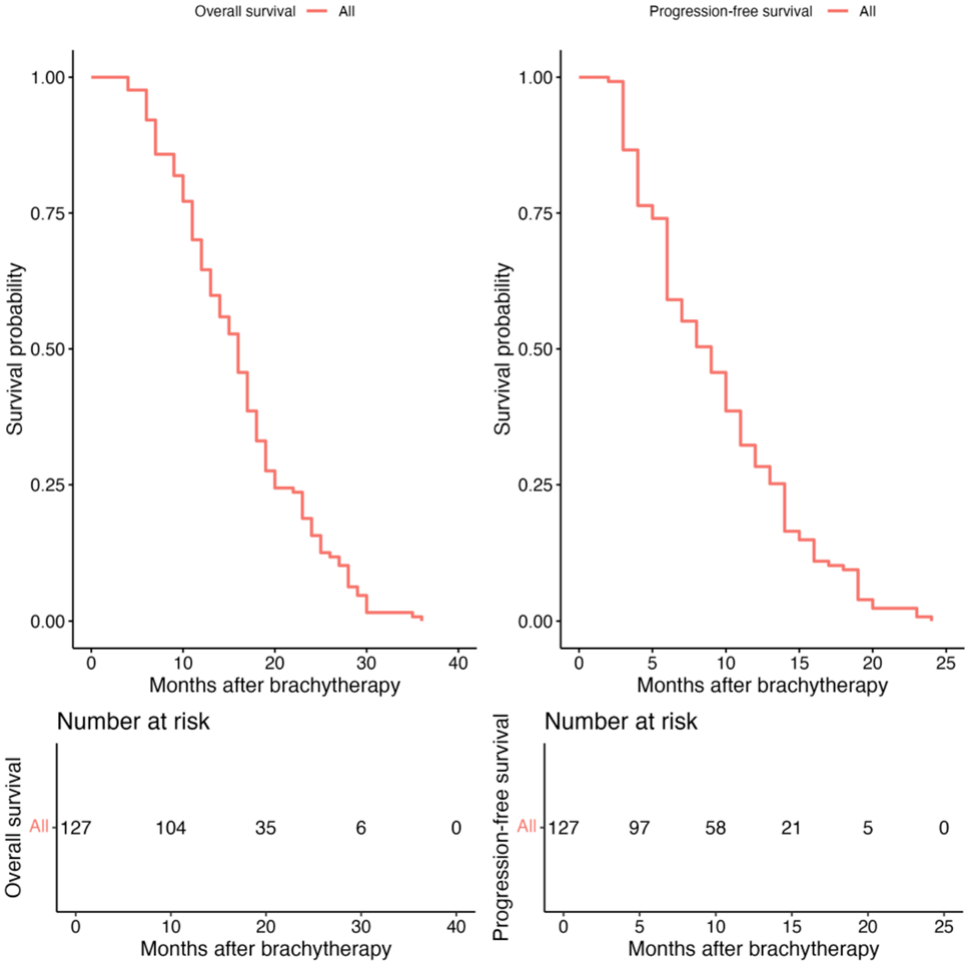
Overall Survival (OS) and Progression-Free Survival (PFS) Analysis for the Entire Cohort

The clinical characteristics and treatment details of the 127 patients are summarized in Table 2. Notably, a total of 262 metastases were treated, with the majority of patients (67.7%) receiving third-line or later therapies. Additionally, 58.3% of patients had liver-only metastases, and 71.7% presented with one or two metastatic foci (Table 2). According to RECIST 1.1, complete response (CR) was achieved in four patients (3.1%), progressive disease (PD) in 30 patients (23.6%), partial response (PR) in 25 patients (19.7%), and stable disease (SD) in 68 patients (53.5%) (Fig. 4). The LC rate at 12 months was 76%. The median total follow-up was 16 months (range: 3–36 months). Median OS was 16 (4–36) months, median PFS was 9 (2–24) months. The 1- and 2-year rates of OS and PFS were 65% (57–73%) and 16% (11–24%) as well as 28% (21–37%) and indeterminate, respectively (Fig. 5). Median time to polimetastatic progression was 14 (14–16) months, median time to next systemic therapy line was 13 (12–15) months (Supplementary Materials (SuppMat) 2 Figure S1, Figure S2) and was accessed from CT-BRT. In 34.6% patients, despite the diagnosis of progression, there was no change in systemic treatment due to the patient’s poor general condition or lack of consent to continue systemic treatment. The Cox proportional hazards regression model investigated the predictive variables modifying OS and PFS (Table 3). Univariate analysis identified metastases outside the liver, specific metastatic sites, DCR, ORR, and RECIST 1.1 response as significant factors influencing OS (Table 3). Metastases outside the liver worsened OS by 1.5-fold (HR = 1.49, *p* = 0.03) with 1-year OS of 53% vs. 73% and 2-year OS of 11% vs. 19%. DCR improved prognosis (HR = 0.42, *p* = 0.001) with 1-year OS of 70% vs. 47% and 2-year OS of 20% vs. 3.3%. Similarly, ORR showed better outcomes (HR = 0.48, *p* = 0.001), with 1-year OS of 76% vs. 61% and 2-year OS of 31% vs. 11%. When RECIST 1.1 response criteria were considered, OS differed significantly (global log-rank *p* = 0.001). The hazard ratio for OS was approximately 15-fold higher for PD (HR = 15.6, *p* = 0.001) compared to PR (HR = 5.36, *p* = 0.024) and SD (HR = 7.61, *p* = 0.006). The 1-year OS rates were 100% for CR, 47% for PD, 72% for PR, and 68% for SD, while the 2-year OS rates were 75% for CR, 3.3% for PD, 24% for PR, and 15% for SD. Although a specific site of metastasis was significantly associated with OS prognosis (global log-rank *p* = 0.047), individual models showed a trend. The presence of additional lung metastases (LuM) worsened OS (HR = 1.42, 95% CI 0.97–2.07, *p* = 0.07) by 1.5 times, followed by additional lymph node metastases (LnM) of the worst prognosis (HR = 1.92, 95% CI 0.96–3.85, *p* = 0.07). One-year OS was 73%, 55%, and 44% for liver-only metastases, LuM, and LnM, respectively. Two-year OS was 19% vs 11% and 11%. In a multivariable analysis, only RECIST 1.1 response was a significant prognostic factor for the OS (Likelihood ratio test *p* = 6e−07; PD—HR = 16.32, 95% CI 3.69–72.22, *p* = 0.0002; PR—HR = 5.84, 95% CI 1.35–25.32, *p* = 0.02; SD—HR = 8.19, 95% CI 1.92–34.93, *p* = 0.004 to CR as reference). In turn, the univariate model revealed that the prescribed dose, Lum and LnM, achieving DCR or ORR response according to RECIST 1.1, and the number of liver-only metastases were relevant for PFS. However, only the presence of metastases outside the liver (LuM) and the achievement of ORR comprised significant factors in the multivariate model (Likelihood ratio test *p* = 4e−04; LuM—HR = 1.47, 95% CI 1.01–2.15, *p* = 0.045 vs liver-only metastases; ORR—HR = 0.46, 95% CI 0.3–0.71, *p* = 0.0005 vs no ORR). The best outcomes regarding PFS were observed when 25 Gy was prescribed in comparison with 15 Gy (HR = 0.46, 95% CI 0.29–0.74, *p* = 0.001; 1-year PFS: 50% (36–69%) vs 13% (5.8–30%). The Kaplan-Maier curves for OS and PFS are shown in SuppMat Figure S3. Noteworthy, the analysis of the optimal cutpoint revealed that the presence of three or more metastases is a cutoff point significantly worsening the OS by over 1.5 times (HR = 0.65, 95% CI 0.44–0.96, *p* = 0.029). Additional OS data from the subgroup analysis of selected variables are provided in SuppMat 2 Figure S4.

**Table 2:** Clinical characteristics of patients included in the study and computer tomography (CT)-guided high-dose rate brachytherapy (BRT) procedure

Variable	Number of patients (%) or median (range)
Age (years)	65 (32–87)
*Sex*	
Male	65 (51.2)
Female	62 (48.8)
*Primary tumor location*	
Rectum	63 (49.6)
Sigmoid colon	23 (18.1)
Descending colon	17 (13.4)
Transverse colon	8 (6.3)
Ascending colon	16 (12.6)
*Number of liver metastases*	
1	37 (29.1)
2	54 (42.5)
3	27 (21.3)
4	9 (7.1)
*KRAS/NRAS mutation status*	
Mutation present	54 (42.5)
Without mutation	73 (54.5)
*Histological grade (G)*	
1	35 (27.6)
2	73 (57.5)
3	19 (15.0)
*Number of lines of systemic treatment before CT-BRT*	
1	6 (4.7)
2	39 (30.7)
3	52 (40.9)
4	26 (20.5)
5	4 (3.1)
*Metastases outside the liver before rOPD*	
Yes	53 (41.7)
No	74 (58.3)
*Lung metastases before rOPD*	
Yes	44 (34.6)
No	83 (65.6)
*Metastases to lymph nodes in the abdominal cavity and/or pelvis before rOPD*	
Yes	14 (11.0)
No	113 (89.0)
Total longest dimension of the metastatic lesion before CT-BRT (cm)	4 (1–8)
The volume of all liver metastases before CT-BRT (cm3)	128 (2–1372)
*No of applicators used*	
1	23 (18.1)
2	37 (29.1)
3	41(32.3)
4	18 (14.2)
5	6 (0.05)
6	0 (0.0)
7	1 (0.01)
8	1 (0.01)
*Dose*	
15 Gy	38 (29.9)
20 Gy	53 (41.7)
25 Gy	56 (44.1)
D 2/3 in uninvolved liver volume (D66) (Gy)	2.1 (0.2–4.4)
*Polimetastatic progression*	
Yes	105
No	22
*Next systemic therapy line after progression*	
Yes	83
No	44

**Table 3:** Univariable Cox Regression Analysis for Prognostic Factors of Overall Survival (OS) and Progression-Free Survival (PFS)

Variable	Overall survival			Progression-free survival		
	HR	95% CI	*p*	HR	95% CI	*p*
*Dose*						
15 Gy	Ref	Ref	Ref	Ref	REF	Ref
20 Gy	1.06	0.69–1.61	0.8	0.78	0.51–1.2	0.3
25 Gy	0.67	0.42–1.07	0.094	0.46	0.29–0.74	0.001
*Metastasis outside of liver*						
No	Ref	Ref	Ref	Ref	Ref	Ref
Yes	1.49	1.04–2.12	0.029	1.46	1.02–2.09	0.038
*DCR*						
No	Ref	Ref	Ref	Ref	Ref	Ref
Yes	0.42	0.27–0.64	< 0.001	0.15	0.1–0.25	< 0.001
*ORR*						
No	Ref	Ref	Ref	Ref	Ref	Ref
Yes	0.48	0.31–0.75	0.001	0.47	0.3–0.72	< 0.001
*Response according to RECIST 1.1*						
CR	Ref	Ref	Ref	Ref	Ref	Ref
PD	15.6	3.56–68.4	< 0.001	14.4	4.84–43	< 0.001
PR	5.36	1.24–23.2	0.024	1.6	0.55–4.64	0.4
SD	7.61	1.81–32	0.006	2.57	0.93–7.12	0.07
*Site of metastasis*						
Liver-only	Ref	Ref	Ref	Ref	Ref	Ref
Liver and lungs	1.42	0.97–2.07	0.07	1.41	0.96–2.06	0.076
Liver and lymph nodes	1.92	0.96–3.85	0.066	1.79	0.89–3.63	0.1
*Number of liver metastases*						
1	Ref	Ref	Ref	Ref	Ref	Ref
2	0.95	0.62–1.44	0.8	0.97	0.63–1.47	0.9
3	1.77	1.07–2.95	0.027	1.93	1.17–3.19	0.01
4	1.03	0.49–2.16	> 0.9	1.24	0.59–2.57	0.6
*Optimal cutoff point*						
One or two	Ref	Ref	Ref	Ref	Ref	Ref
Three and more	1.55	1.05–2.29	0.029	1.17	0.8–1.72	0.4

### Treatment-related toxicity

The most frequently observed acute side effect of CT-BRT was a G1/G2 increase in transaminases in 28 (22%) patients, followed by G1/G2 pain at the injection site, which was reported in 25 (20%) patients. Additionally, twelve patients (9.5%) experienced G1/G2 nausea within one week following the procedure. All of these side effects were transient and managed effectively with pharmacotherapy. G1/G2 infections at the injection site occurred in 5 (4%) patients, resolving upon antibiotic administration. An occurrence of significant G1/G2 bleeding necessitating red blood cell transfusion (RBC) developed in 3 (2.4%) of patients within 24 h of the CT-BRT. No surgical intervention was required for the entire cohort. Four percent of acute G3 complications, involving pain at the injection site and an increase in transaminases or total bilirubin, were resolved within the initial 3 months post-procedure with pharmacological treatment. Notably, no late toxicity relating to liver CT-BRT was reported during the follow-up period. (Table 4).

**Table 4:** Treatment-related acute toxicity according to common terminology criteria for adverse events 5.0

Type of complications	G1/2	G3
Pain at the injection site	25 (20%)	3 (2.4%)
Increased level of transaminases	28 (22%)	1 (0.8%)
Increased level of total bilirubin	19 (15%)	1 (0.8%)
Infection	5 (4%)	0 (0%)
Significant bleeding	3 (2.4%)	
RBC transfusion	3 (2.4%)	
Surgery	0 (0%)	0 (0%)
Pneumothorax	0 (0%)	0 (0%)
Nausea/vomiting	12 (9.5%)	0 (0%)

## Discussion

Our study including 127 with 262 liver metastases, heavily pretreated with systemic therapy, showed that CT-BRT achieved 76.4% disease control (CR + PR + SD) at 6 months. More than 60% of patients with CRC develop metastases during their disease [24]. Genomic profiling enabled the selection of the most effective systemic therapies, extending the potential survival of those patients. Despite modern treatments achieving 1-year, 3-year, and 5-year overall survival rates of 70–75%, 30–35%, and 14–23%, respectively [25], the longest survival is seen in only 5% of patients with mismatch repair deficiency (MMR-D) or microsatellite instability-high (MSI-H) profiles [26]. The CRC patients often develop liver metastasis [27]. The mainstay treatment in that setting is surgical resection. Only 10–30% of patients can be qualified for this procedure due to comorbidities or unwillingness to undergo surgery [12]. It is worth mentioning that postoperative complication rates after metastasectomies account for 11.4–15% [28–30]. Almost half of patients with metastatic CRC are within KRAS/NRAS/BRAF wild-type molecular profiles. Patients with KRAS/NRAS sequence variation account for 35–45% [25]. For those two most frequently present molecular subtypes, the median OS time after progression on the first line of systemic treatment is 8–18 months; after the second line, it is 4–12 months, and 2–8 months after the third line [25]. Patients harboring BRAF sequence variations present even shorter OS. Considering the abysmal prognosis of patients showing progression after the first and subsequent lines of systemic treatment, it is crucial to explore the option of using metastasis-directed therapy, especially in the case of OMD. By doing so, we can extend the time until further progression and provide additional time before the following line of systemic treatment is introduced. Ultimately, this approach could translate into increased PFS and OS for these patients. The OMD is an umbrella term, and it has already been addressed that nine different clinical scenarios can be defined according to ESTRO/EORTC recommendations [16, 17]. It is essential to distinguish precisely those scenarios in OMD treatment analyses to facilitate comparing outcomes of different modalities that can be proposed and find a subgroup of patients that benefit the most from MDT.

While resection is considered the gold standard, nearly 80% of patients with liver metastases are classified as inoperable due to patient-specific factors or as unresectable due to technical limitations [31]. Unfortunately, no prospective randomized trial results address the treatment of OMD CRC patients. MAVERRIC trial was a multicenter prospective cohort study in which results comparing stereotactic microwave ablation (SMWA) with metastasectomy were presented. Patients with no more than five liver metastases, no larger than 3 cm, were qualified. According to the direct OMD clinical scenario definition, there was no information, assuming patients with synchronous liver metastases without progression were also qualified. Compared to metastasectomy, the 3-year and 5-year OS rates after SMWA were 78–76% (*p* = 0.861) and 56% to 58%, respectively. What is worth mentioning is the fact that overall and major complications were lower after SMWA (67% and 80% decrease, *p* < 0.01) [32]. The SBRT, is the most frequently used radiotherapy modality to treat secondary liver malignancies. A systematic review of its efficacy in CRC patients with liver metastases gathered data from 18 studies. It was shown that 1- and 2-year OS were 67.18% and 56.5%, respectively. Median PFS and OS were 11.5 and 31.5 months. Grade 1⁄2 toxicities and G3 liver toxicity were present in 30.7% and 8.7% of patients [33]. The recent meta-analysis summarizing data of 3101 patients underscored favorable outcomes with liver metastasis SBRT, demonstrating pooled 1- and 3-year local control rates of 85% and 68%, respectively [34]. This method has been introduced in the newest ASCO guidelines for patients with metastatic CRC [35].

Although widely used, SBRT faces challenges in treating liver metastases, due to significant liver motion, necessitating adding CTV and PTV margins. A systematic review shows intra-fraction variability of up to 9.7 mm with free breathing, while breath hold, and abdominal compression limit it to within 3 mm [36]. This limitation is not an issue in CT-BRT where catheters are inserted firmly into the liver and thus without PTV expansion allow for the sparing of healthy tissue. For large metastases (> 3 cm), SBRT presents favorable outcomes as compared to radiofrequency ablation (RFA), SMWA, cryoablation, or transarterial embolization [34]. But still, for large metastases, the dose of SBRT, which is often restricted to minimize toxicity, is constantly reported as the main predictor of LC [34]. This issue arouses as an advantage of CT-BRT as dose near catheters exceeds 1000 Gy and presents step dose fall outside of target favorable as compared to SBRT [37]. In our study, the dose was not restricted due to the volume of the lesion, but only because of the proximity of the OARs.

In the current study, we have qualified 127 patients with CRC with rOP in the liver only, for which we have performed MDT with CT-BRT. To our knowledge, this is the largest reported cohort of CRC patients treated with liver CT-BRT or SBRT in the literature. The most extensive series, by Ricke et al. and Colletoni et al., involved 73 and 80 patients with 199 and 179 liver metastases, respectively [38, 39]. To our knowledge, no previous studies on liver metastases radiotherapy, including outcomes and toxicity, have implemented the modern ESTRO/EORTC definition of OMD, making our analysis unique. A summary of studies investigating the treatment of CRC liver metastases using brachytherapy is presented in Table 5 [13, 38–41].

**Table 5:** Previous studies involving liver brachytherapy for colorectal cancer liver metastases

Author	No of patients with CRC liver metastases	Prescribed dose	Outcomes	Treatment-related toxicity
Colletini et al. [39]	80 (100%)	15–20 Gy	1-y LRFS-88.3% 2-y LRFS-81.2% 3-y LRFS-68.4% 1-y OS-87.6% 2-y OS-68.4% 3-y OS-41.6% mPFS-6 months	No major according to Society of Interventional Radiology (SIR) standards
Ricke et al. [38]	73 (100%)	15–25 Gy	15-m LRFS-(< 25 Gy)-75% 15-m LRFS-(> 25 Gy)-95% mOS-23.4 months 1-y PFS (25 Gy)-50% 1-y PFS (15 Gy)-13%	5 (6.8%) major • 2 (2.5%) occult bleeding needing blood transfusion, • 2 (2.5%) gastric ulcer, • 1 (1.1%) recurrent pleural effusion needing pleurodesis
Walter et al. [40]	54 (51%)	25 Gy	6-m LRFS-84.1% 1-y LRFS-50.6% 6-m OS-95.2% 1-y OS-72.4% 6-m PFS-40.3% 1-y PFS-11.2%	4 (3.6%) major, • 2 (1,8%) abscess of the liver • 1 (0.9%) segmental cholestasis, • 1 (0.9%) major bleeding event resulting in hematoma
Kieszko [13]	46 (75.4%)	20 Gy (15–25)	6-m LRFS-88.7% 1-y LRFS-70.7% 6-m OS-96.7% 1-y OS-79.6% 6-m PFS-78.1% 1-y PFS-53.8%	No > G2 CTCAE 4.0 toxicity, G2- 4 (7%)- pain at the injection site
Tselis et al. [41]	14 (31.4%)	20 Gy (7–32)	6-m LRFS-89% 1-y LRFS-73% 18-m LRFS-63%	In the whole cohort (41 pts.) 3 (5%) major—9 (15.2%) minor

One of the most significant advantages of CT-BRT, above SBRT or interventional radiology procedures, is that large tumor sizes can be easily qualified and treated. The two most extensive series presenting liver brachytherapy results only with CRC patients involved metastases of median size of 5 cm (1–13 cm) and 2.85 cm (0.8–10.7) [38, 39]. In our cohort, the median size of the tumor was 4 cm (1–8), and the median tumor volume was 128 cm^3^ (2–1372). Our maximal tumor volume was similar to Tselis et al., where patients with a median tumor volume of 84 cm^3^ (38–1348 cm^3^) were included [41]. In our series, a 1-year LC of 76% was achieved, consistent with the results of other authors. Ricke et al. tested three dose levels of 15, 20 Gy, and 25 Gy, presenting 15-month local control (LC) rates of 75% [38]. Colletoni et al., with a median prescription dose of 19.1 Gy (15–20), presented 1-year and 2-year LC rates of 88.3% and 81.2%, respectively [39]. The authors reported 1-year and 2-year OS of 87.6% and 68.4%, respectively. In our series, a more divided analysis of outcomes according to RECIST 1.1 criteria was performed. Patients who achieved ORR presented the best 1-year and 2-year OS results of 76% and 31%, respectively. What is worth mentioning is that achieving CR resulted in 1-year and 2-year OS of 100% and 75%, respectively. The univariate model revealed that radiation dose, additional metastases outside the liver, number of liver metastases, and type of response according to RECIST 1.1 were relevant for PFS prognosis. In the multivariable model, only achieving ORR and the presence of non-liver metastases were significant prognostic factors for PFS. Doses of 25 Gy led to the best outcomes, with 1-year PFS of a median of 1-year PFS: 50% vs 13% for the cohort treated with 15 Gy. This is consistent with previous reports mentioning that a dose of 25 Gy and more led to 95% LC at 1 -year [38].

We are aware of only one study presenting results of liver metastases SBRT employed for oligoprogression of CRC [42]. Nineteen patients with oligoprogression, defined as up to 5 new and/or growing metastases with other stable lesions, were included. Cyberknife platform was used, and patients had 2–4 fiducial markers implanted before SBRT. The median prescribed BED10 dose was 105.6 Gy. Median PFS was 6.8 months (95% CI 5.71–7.89 months). One-year PFS was 22.3% (95%CI 9.4–52.9), and 1, 2, and 3-year OS of 75.3% (95%CI 56.93–99.6), 62.7% (95%CI 43.04–91.5), 21.5% (95%CI 6.63–69.8), respectively [42]. Our cohort achieved a median OS of 16 months and a median PFS of 9 months. Patients were already heavily treated with systemic therapy and the median number of systemic therapy lines used before CT-BRT was 3 (1–5). We have to bear in mind that those patients’ survival and progression time is worsening with every other line of systemic treatment [25].

In our cohort, mainly G1/G2 treatment-related toxicity was seen. Only 4% of patients had acute G3 toxicity, which included pain at the injection site, hyperbilirubinemia or hypertransaminasemia. None of the patients needed surgical procedures because of toxicity or peri-procedure complications. In our previous report, we showed that, within 6 months of CT-BRT, levels of biochemical liver function markers do not correlate with physical parameters such as Dmax, D 1/3, D 2/3, D 50%, D 10 cm^3^, D 100 cm^3^, D 500 cm^3^, D 100%, D 90%, or liver volume [15]. However, transient transaminasemia or hyperbilirubinemia after CT-BRT depends on the irradiated tumor volume (TV) [15]. That indicates a specific TV at which this type of toxicity might not be acceptable, especially when the patient gets potentially hepatotoxic systemic therapy. According to reports, the major complication rate after brachytherapy is 0–5%, including treatment of metastases up to 14 cm [38, 39, 42–44]. It was previously reported that after SBRT, G3 and G4 toxicity can account for 21.5–23.2% and 7.5%, respectively [45, 46].

Reports have compared dosimetric characterization of LINAC-based SBRT plans with CT-BRT, showing that CT-BRT offers optimal dose distribution and better protection of organs at risk [18, 37].

The main limitation of this study is its retrospective character and lack of randomization. Another limitation of this study is that only RECIST 1.1 criteria were used. Other researchers have demonstrated that PET/CT with 18F-FDG, utilizing the PERCIST classification, provides the highest sensitivity in response evaluation after SBRT for liver metastases [47]. When CT is used, some studies suggest that Choi criteria might be better than RECIST 1.1, but it needs future evaluation on larger cohorts [21, 48]. Finally, response evaluation based on DW-MRI can be one of the best options in the qualitative and quantitative assessment of treatment response after CT-BT of CRC metastases in the liver [21].

## Conclusions

We demonstrated that using CT-BRT to treat liver metastases in CRC patients with rOPD can lead to favorable outcomes, even in those heavily pretreated with systemic therapies. Importantly, this approach offers a favorable toxicity profile. For patient’s ineligible for or declining surgical resection, CT-BRT remains a viable and effective MDT in this clinical setting.

## Supplementary Information

Below is the link to the electronic supplementary material.

Supplementary file1 (DOCX 25 kb)

Supplementary file2 (DOCX 1373 kb)

## Abbreviations

CT-BRT: Computer tomography (CT)-guided high-dose rate brachytherapy (BRT)
CTV: Clinical Target Volume
CR: Complete response
CRC: Colorectal cancer
DCR: Disease control rates
EBRT: External beam radiotherapy
EORTC: European Organisation for Research and Treatment of Cancer
ESTRO: European Society for Radiotherapy and Oncology
FU: Follow-up
GTV: Gross tumor volume
LC: Local control
LnM: Lymph node metastases
LuM: Lung metastases
MDT: Metastasis-Directed Therapy
OARs: Organs at risk
OMD: Metastatic disease
ORR: Objective response rate (ORR)
OS: Overall survival
PD: Progressive disease
PET/CT: Positron emission computer tomography
PFS: Progression-free survival
PR: Partial response
PTV: Planning target volumes
RO: Radiation oncologist
rOP: Repeat oligoprogression
SD: Stable disease
SBRT: Stereotactic body radiotherapy
TV: Tumor volume
