# Supplementary material for: Comprehensive cohort study: computer tomography-guided high-dose rate brachytherapy as metastasis-directed therapy for liver metastases from colorectal cancer in repeat oligoprogression
Source: Radiol Med. 2025 Mar 13;130(5):694–705. doi: 10.1007/s11547-025-01988-y (PMC12106498; doi:10.1007/s11547-025-01988-y)
Supplement: Supplementary file 2 — Supplementary file2 (DOCX 1373 kb) [file 11547_2025_1988_MOESM2_ESM.docx]

Table S1. Dose constraints for liver brachytherapy planning.

| Organ | Dose (D) or volume (V) constrains |
| --- | --- |
| Uninvolved Liver | V10Gy < 700cc  D66% < 10Gy |
|  |  |
| Spinal Cord | Dmax < 15Gy  D1cc < 14Gy |
|  |  |
| Bowel | Dmax < 15Gy |
|  |  |
| Gallbladder | Dmax < 20Gy |
| Great Vessels | D1cc < 27Gy |
| Ribs | D1cc < 23Gy |
| Heart | Dmax < 22Gy |
|  |  |
| Kidneys | Dmean < 6Gy  D1cc < 18Gy  D200cc < 10Gy |
|  |  |
|  |  |
| Duodenum | D1cc < 15Gy |
| Stomach | D1cc < 15Gy |
| Esophagus | D1cc < 15Gy |

Figure S1

Kaplan-Meier curve for time to next systemic therapy line following brachytherapy


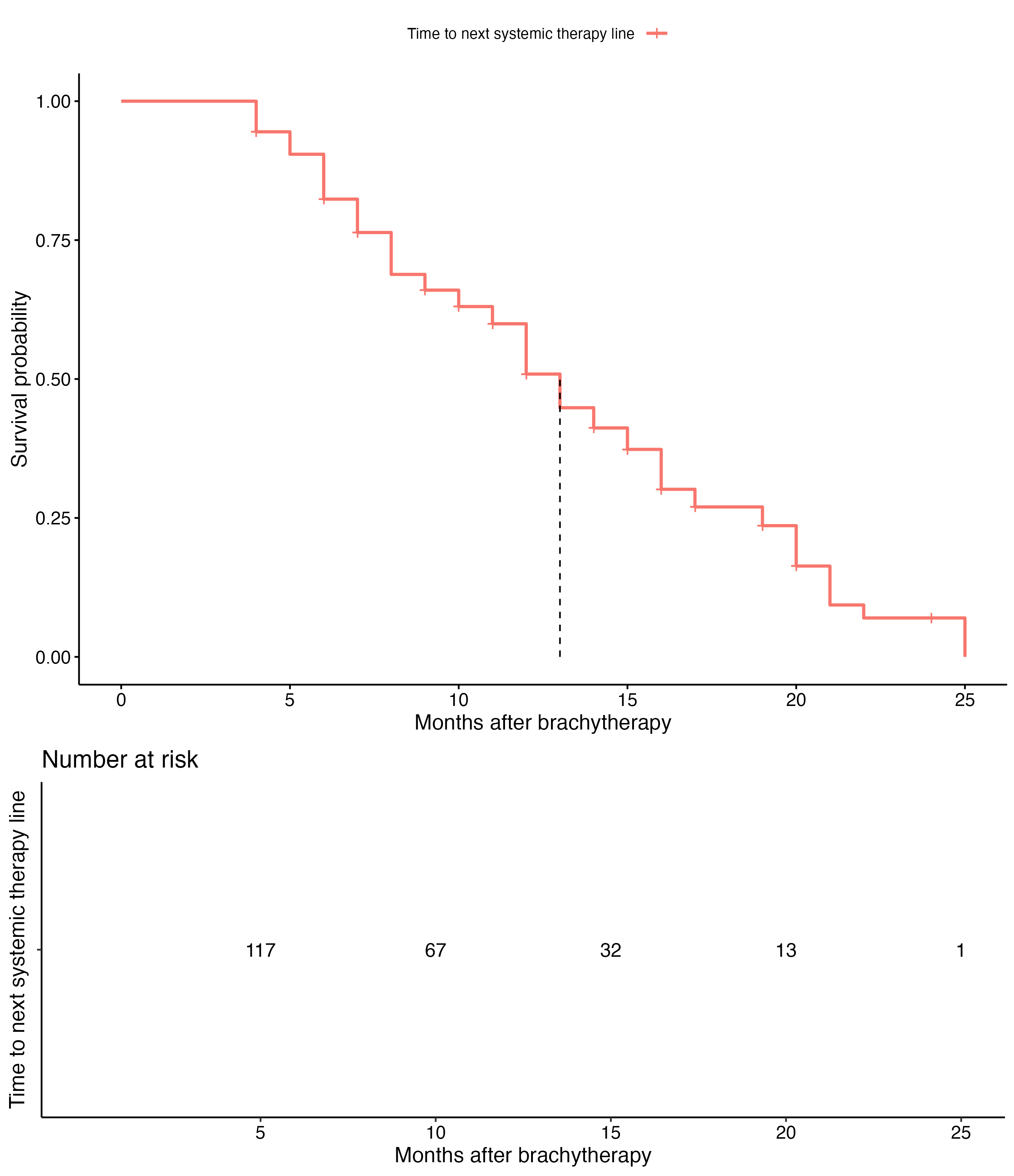


Kaplan-Meier survival analysis showing the probability of delaying initiation of the next systemic therapy line in patients following brachytherapy over time. The x-axis represents the months after brachytherapy, while the y-axis denotes survival probability. The dashed line indicates the median time to the next systemic therapy line. Below the plot, the "Number at risk" table illustrates the number of patients remaining in the analysis at specified time intervals. The analysis encompasses data from 117 patients at the start of follow-up.

Figure S2.

Kaplan-Meier Curve for Time to Polymetastatic Progression Following Brachytherapy


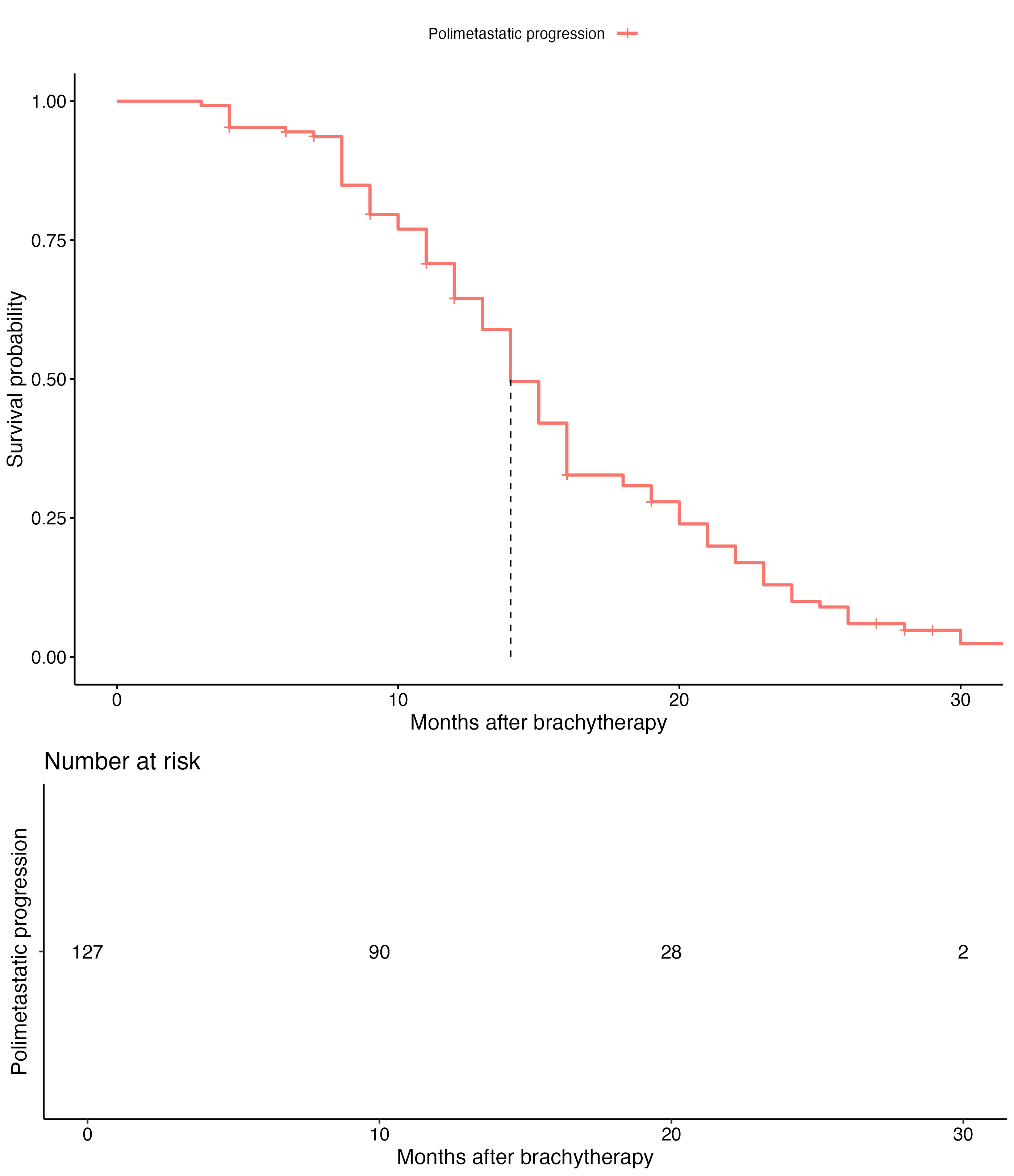


Kaplan-Meier survival analysis illustrating the time to polymetastatic progression in patients following brachytherapy. The x-axis represents the months after brachytherapy, and the y-axis shows the survival probability without progression to a polymetastatic state. The dashed line marks the median time to polymetastatic progression. Below the graph, the "Number at risk" table details the number of patients still at risk at specific time intervals. A total of 127 patients were included in this analysis at the start of follow-up.

Figure S3.

Kaplan-Meier Curves for Progression-Free Survival (PFS) and Overall Survival (OS) Following Brachytherapy

Kaplan-Meier survival analysis depicting both overall survival (OS) (Figure 3a) and progression-free survival (PFS) (Figure 3b) in patients following brachytherapy. The x-axis represents the time in months after brachytherapy, while the y-axis shows the probability of survival. Figure S4 Survival analysis of subgroups of patients

A


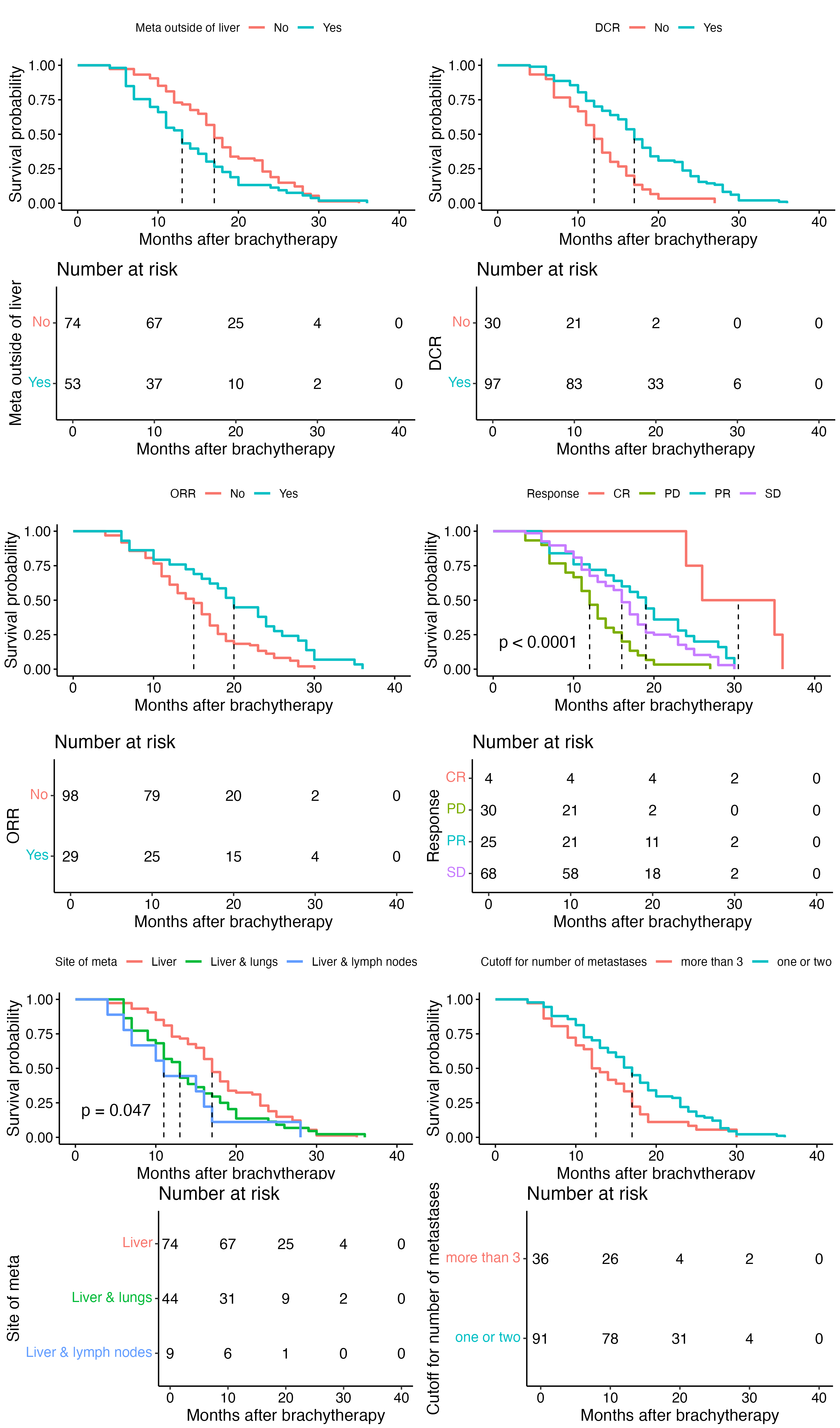


B

F

C

E

D

The figure presents a set of Kaplan-Meier survival curves for 127 patients with liver-related oligoprogressive disease (rOPD) treated with brachytherapy. These curves analyze survival based on several clinical variables: From the left top : A) Meta outside of Liver: Compares survival between patients with metastases confined to the liver (n=74) and those with extra-hepatic metastases (n=53), B) DCR (Disease Control Rate): Illustrates survival differences between patients with disease control (n=97) versus those without (n=30), C)ORR (Overall Response Rate): Shows survival based on response rates, with a division between responders (n=29) and non-responders (n=98), D) Response Types: Details survival outcomes for different types of tumor responses: complete response (CR, n=4), partial response (PR, n=25), stable disease (SD, n=68), and progressive disease (PD, n=30), E) Site of Metastases: Compares survival across patients with metastases only in the liver (n=74), liver and lungs (n=44), and liver and lymph nodes (n=9), F) Number of Metastases: Distinguishes survival rates for patients with more than three metastases (n=36) versus those with one or two metastases (n=91). Each panel includes a 'Number at risk' table, indicating the count of patients remaining in the analysis at specific time intervals (0, 10, 20, 30 months), providing insights into patient attrition over time. If differences were significant p values are shown.
